# Report on the 2013 European Multidisciplinary Cancer Congress—ECC 17, Amsterdam, 27 September–1 October 2013: nursing highlights

**DOI:** 10.3332/ecancer.2013.367

**Published:** 2013-10-24

**Authors:** Rosario Caruso, Cristina Di Pasquale, Danuta Lichosik, Federica Dellafirore, Francesco Pittella

**Affiliations:** 1 Nursing Degree Course, University of Milan, IRCCS Policlinico San Donato, Milan 20097, Italy; 2 Division of Urology, European Institute of Oncology, Milan 20141, Italy; 3 Operating theatre, European Institute of Oncology, Milan 20141, Italy

**Keywords:** oncology nursing, 17th ECCO - 38th ESMO - 32nd ESTRO European Cancer Congress, conference highlights

## Abstract

The European Cancer Organisation (ECCO) was founded on the ideas of the former Federation of European Cancer Societies (FECS). The ECCO was officially announced at the European Cancer Conference in Barcelona in September 2007, replacing the FECS as a dynamic new entity. Through its members, the ECCO represents the interests of over 50,000 professionals in oncology. The ECCO continues to expand its outreach and education through its prestigious biennial series of Congresses. This report highlights the nursing contributions at the seventeenth ECCO Congress in Amsterdam.

At the congress, there were more than 17,000 professionals involved in the struggle against cancer. A record number of abstracts (3306) were submitted, almost 40% more than the 2011 conference. Related topics during nursing sessions were often aimed at investigating the meaning of the multidisciplinary approach and what it implies for daily practice under different profiles. The debates showed that the multidisciplinary approach primarily means ‘new challenges’ for all the practitioners involved. The main challenge for nurses is to meet the needs of a rapidly changing society with some European peculiarities, such as the ageing population, the escalating costs of healthcare in a period of economic crises, fast changing treatments, changes in cancer services and the way nurses deliver care, and multidisciplinary empowerment as a modern concept of care. In this landscape, we also have to consider that cancer often becomes a chronic disease with an increasing number of treatment lines, an increasing number of survivors, and more conscious and exigent patients. We also have to consider the importance of diversity in cancer care.

## Introduction

The paramount purpose of ECCO 17 was once again to combine the united efforts of all partner organisations to continue positioning multidisciplinarity as the way to best improve the prevention of cancer and the diagnosis, treatment, and care of cancer patients. The recognised multidisciplinary setting of the Congress provided ideal surroundings for participants to leverage knowledge, promote education, and build awareness about oncology – placing the patient at the heart of all the efforts and discussions. In this context, the nursing sessions were developed. Major issues that were highlighted include [1]:

– Exploring complexity and special needs in specific cancers
– Advanced nursing roles
– Young people facing cancer
– Cancer pain management barriers: can we overcome them?
– Impact of cancer on patients and families
– Palliative care in 2014
– Multidisciplinary collaboration: reflections on the past, thinking ahead for the future
– Improvements in cancer nursing care
– Management of toxicities related to chemotherapy and targeted therapy
– Hope across cancer care
– Survivorship and follow-up
– Cancer surgery and nursing

## News since ECCO 2013

The opening ceremony gave particular importance to new discoveries, looking at different ways to pump up the body’s natural defences and strip away the cloak of invisibility that cancer cells use to evade our immune system, considering that targeted cancer drugs already use the immune system to home in on cancer cells. Trials are also underway on a new generation of therapies that work directly on the immune system itself. In this landscape, the importance of strengthening the multidisciplinary approach has emerged. According to the European Oncology Nursing Society (EONS) president Erik van Muilekom, we have to consider every patient to be unique, which challenges us to provide personalised care. A current field of progress has discovered the unique qualities of individual tumours, the natural result of which is increasingly personalised cancer therapy. Accordingly it is crucial to take a multidisciplinary approach to care, to think outside the box, and to recognise each other’s qualities and competencies in order to best respond to each individual patient’s need.

## Exploring complexity and special needs in specific cancers

Topics in this session included the impact of cancer and an exploration of the complexity and special needs of specific cancers, particularly regarding cervical cancer, head and neck cancer (HNC), and lung cancer. Hughes examined the changes recommended in the NCSI (National Cancer Survivorship Initiative) document in relation to the care of women with cervical cancer, including the use of formalised models of assessment and care planning to encompass holistic needs that include considerations such as access to work and childcare; templates for treatment summaries that support primary care in the management of women post treatment; the provision of a health and wellbeing event to facilitate the transition from treatment to living with and beyond cancer, including the role of physical activity and a healthy lifestyle; risk stratification of follow-up to ensure women with low-risk disease have information about the effects of treatment, what to look for in the event of recurrence; access to services and the encouragement to self-manage; and ensuring that women with complex needs get more intensive and appropriate support.

Wells reported evidence from a recent systematic review of qualitative research on living with HNC and on focus groups with patients and careers that highlight the ways in which treatment and care experiences can affect their quality of life (QoL). Findings from a cross-sectional survey conducted in Scotland will be used to illustrate the most prominent concerns, including QoL issues and unmet needs expressed by survivors in the first five years after treatment.

Serena spoke about lung cancer, the most frequent cancer affecting both men and women in the world. She highlighted a review of innovative models of care and emerging trends for nurses as members of a multidisciplinary team caring for lung cancer patients and their families. Current evidence related to the use of non-pharmacologic interventions aimed at improving QoL in lung cancer patients was provided. The importance and promise of several approaches for patients with lung cancer were identified: a key role for the assessment of early needs; a role for LCN (lung cancer nurse specialist) consultation at diagnosis, through treatment and continued through follow-up care; and targeted therapeutic education to promote self-management.

## Advanced nursing roles

These issues were argued during presented papers where advanced nursing roles were analysed. The debates emphasised the increasingly important role of mobile devices and technologies in the modern concept of nursing, considering both education and care. This ranged from the use of different types of mobile devices and technology, including telephone consultations in nursing practice, both during the treatment for easy access to clinical information and to empower the post-treatment follow-up as well as the nursing training. The creation of a nursing PICC (peripherally inserted central catheter) Team was discussed by Bianchi. The project presented, started on the initiative of Swiss nurses, has been developed and consolidated thanks to a multidisciplinary collaboration. The PICCs introduction has promoted a development of nurse professional competencies, and patients have been shown to have a higher chance of receiving the correct vascular device in line with their clinical needs.

Telephone follow-up as a strategy to improve the adherence to treatments of cancer patients was reported by Rihuete. The author explained that treatment adherence is a subject of great importance in oncology because it impacts on the effectiveness of treatments, patient safety, and health costs. An early identification of poor adherence will help to prevent treatment failures. An experimental design was used to evaluate telephone follow-up intervention, and participants were randomised into a control or a treatment group. Rihuete explained that her results show that systematic information by telephone follow-up improves the adherence to treatment of cancer patients.

The experience of using a telephone for women newly diagnosed with breast cancer was shared by Pihlmann, providing valuable knowledge about newly diagnosed breast cancer patients’ informational needs. Elfrink has reported the implementation of nursing research in oncology nursing at the Erasmus MC Cancer Institute, where good results have been achieved within a few years. Milani spoke about a multicentre project funded with support from the European Commission, aimed at developing a new approach to training using mobile devices to extend learning activity into daily practice. An English study aimed at investigating the nursing role within nurse-led oncology clinics was presented by Farrell. There has been a rapid expansion and development of nursing roles and responsibilities in oncology, but little understanding of how these roles are enacted not their impact on patient experience and outcomes. Nurse-led clinics have adopted a medical model of doctor–nurse substitution, which may have led to reduced emphasis on nursing skills and compassionate care.

## Young people facing cancer

Cancer is the leading cause of disease and death in developed countries, taking into consideration that in teenagers and young adults, it is uncommon, and overall five-year survival rates are an impressive 80%. Fern inspired the audience by discussing the evidence of a prolonged diagnostic journey for young people with cancer. Survival for some cancers, notably bone sarcomas, in young people is less than in children and in older adults. This may be related to a prolonged period of diagnosis, differences in cancer biology, lesser involvement in clinical trials, and place of care. Young people themselves, often in conjunction with secondary healthcare professionals, frequently point to primary care as a simplistic solution to improving the diagnostic journey. However, there are multiple components of the diagnostic pathway where actual or perceived ‘delays’ can occur. These range from appraisal and recognition of potential symptoms by young people, accessing appropriate healthcare services, referral from primary care and also system processes within secondary care.

Eilertsen presented a Norwegian case study that included 50 children and adolescents diagnosed with cancer. Data for the study were collected using The Inventory of Life Quality in Children and Adolescents (ILC) questionnaire and the Revised Questionnaire to Assess Health-Related Quality of Life in Children and Adolescents (KINDL) (parent and self-reports). Data were also collected from the medical records of children surviving cancer; the study sought any evidence of somatic late effects and psychological symptoms. In summary, when planning long-term follow-up care and rehabilitation of children and adolescents with cancer, especially for survivors with brain tumours and with late effects, it is important to take into account their subjectively perceived and proxy reported QoL, in addition to their psychological problems and psychosocial function.

Smith spoke about the importance of delivering age appropriate care for teenagers and young adults with cancer, and Thompson presented an Australian model of care in development for young cancer survivors, discussing the current model of Survivorship Care being piloted and some preliminary learning to date; specifically in relation to transferability, sustainability, and the unique, emerging post-treatment survivorship issues for young people in Australia.

## Cancer pain management barriers: can we overcome them?

Pain management remains a challenge for patients with cancer. The reasons for this are numerous, among them attitudinal barriers that have been found to negatively affect the quality of pain management and, in turn, QoL. These attitudinal barriers include fatalistic beliefs about cancer pain, as well as fear of addiction, concerns about tolerance and side effects, concerns that strong pain medications mask changes in one’s body and harm the immune system, the belief that ‘good patients’ do not complain about pain, and the belief that reports of pain may distract physicians from treating the underlying disease. Such barriers are prevalent among patients, family members, the general public, and healthcare professionals including physicians and nurses. Piskorjanac presented a report on nursing interventions. Nurses are in a prime position to allay the patient fears, to educate patients, and to ensure that effective pain control is integrated into the complete programme of healthcare provided for the patient. In particular, when nursing patients for whom the prospect of cure is remote or non-existent, putting pain control at the forefront of palliative care is crucial. The debate highlighted that in overcoming barriers to the treatment of pain, nurses must use a cultural approach, taking into account the patient’s personal context.

## Impact of cancer on patients and families

This article session started with Van Humbeeck’s presentation of a qualitative study using loosely structured interviews to elicit accounts of twenty parental caregivers under the age of 70. Van Humbeeck explained that ‘Suffering in silence’ emerged as a descriptor of their experiences. Older parents’ situations can be envisioned as facing a delicate balancing act between shielding their child from pain while being shielded by their child and between being involved with care while keeping an adequate distance. Faced with the adult child’s illness and possible death, older parents are confronted with overwhelming feelings, which are often underestimated or downplayed. Healthcare professionals can play a pivotal role in protecting the autonomy and privacy of adult children while practicing family-centred principles. A cancer diagnosis has a physical and emotional impact on the patient; however, during this time, the family caregivers providing support are often at risk of physical, emotional, and practical problems themselves.

Coyne presented an interpretive study investigating oncology nurses’ family assessment practices across cancer care inpatient and day-treatment areas of three metropolitan hospitals in South-east Queensland. The study participants highlighted that the use of a structured approach would improve their family assessment techniques, suggesting a set of questions to ask the patient and family, which might help the less-experienced oncology nurse. Another interesting issue regarded the QoL assessment in patients with HNC. Verschueren reported a pilot study comparing touch screen technology and paper questionnaires. Using a descriptive qualitative design, Semple presented findings wherein open communication is essential for children whose parents have cancer, noting that parents need support and resources to promote family coping when they are diagnosed with cancer. Coolbrandt reported on qualitative research aimed at improving our insight into the experiences professional care needs of patients with high-grade glioma, and the unique needs of their family caregivers: both patients and caregivers express the need for the professional care community to demonstrate increased willingness to listen to their issues, as well as better information for patients and carers. Families also need to feel that healthcare professionals are aware of the seriousness of the diagnosis, and that they take this into account in their approach of both patient and family caregiver.

## Palliative care in 2014

Palliative sedation, a medical intervention aimed at relieving intractable suffering at the end of life by inducing decreased awareness of symptoms, has become a substantial practice in end-of-life care. Using a quantitative questionnaire and additional qualitative interview, Swart researched the last patient for whom Continuous Palliative Sedation (CPS) until death had been used. Nurses, more often than physicians, indicated that patients were anxious prior to the start of continuous sedation, and they more often mentioned pain as the decisive indication for starting CPS. Compared with doctors, nurses less often felt pressure from patients or relatives to start CPS. Different experiences of physicians and nurses with CPS in clinical practice may be a reflection of their different roles. While proportional sedation is usually described as a dose of sedatives titrated to relieve refractory symptoms, this study demonstrated that in practice, proportional sedation is used in ways that are highly specific to the needs and preferences of patients and their relatives. The place of rehabilitation in cancer and survivorship as an initiative to promote QoL is well established. A core message in relation to palliative care rehabilitation is that it should meet patient goals, be provided by a multidisciplinary team and be cognisant of the core issues of independence and functional mobility.

## Multidisciplinary collaboration. Reflections on the past, thinking ahead for the future

It emerges that nurses have to strengthen a network to improve collaborative, international oncology nursing research and to foster multidisciplinary collaboration among the different professional organisations, such as between EONS and ECCO, ESMO, ESSO, ESO, ESTRO and SIOPE. The field of care is moving away from entrenched habits and cultures wherein healthcare professionals formerly only considered the areas of care in which they operated. The era of multidisciplinary collaboration will take into account the interactions between different spheres of healthcare. During this session, presidents of the above-mentioned organisations described their policies and relationships with the field of cancer nursing, crossing the history of the various organisations. The president of ESSO, Professor Peter Naredi, emphasised that we often think that multidisciplinary is the future, but that this future is already present.

## Improvements in cancer nursing care

The number of individuals who survive after a diagnosis of cancer is growing steadily, and cancer survivorship does not come without a cost. This was the background against which Fitch delivered a discussion on implementing the use of survivorship care plans. The purpose of the presentation was to create and implement a sustainable survivorship care plan approach. The objective was to learn about the barriers to implementing survivorship care plans in the Canadian healthcare environment. The project illustrated that the use of survivorship care plans in Canada was feasible. Survivors reported that the plans helped them to understand the next steps in their cancer journeys. Each jurisdiction reported that the following elements were important factors for successful implementation of survivorship care plans: leadership, teamwork, and collaboration, tailoring the care plan, education and training, communication and dissemination, and conceptualisation of survivorship. In all cases, the projects streamlined the transition from treatment to survivorship, and significantly increasing the capacities of patients and healthcare providers to address this gap in care provision. It is possible that the community, cancer centre, and on-line modalities could all work as complementary systems to bridge the gap for cancer survivors, offering care plan services at a variety of times and places to suit the range of patients’ needs along the survivorship trajectory.

Paterson presented a study with two phases: phase one was a prospective longitudinal survey (*N* = 74) and phase two captured real-time patient-reported outcome measures in the form of an electronic behavioural diary. A sub-sample from the prospective, longitudinal survey (*n* = 12) completed the electronic behavioural diary in the weeks following treatment. A Research Steering Group, formed of patients with prostate cancer and clinicians, informed the development of the electronic behavioural diary. This innovative study demonstrates the acceptability of e-health technology in prostate cancer survivors and may provide a platform to deliver a supported self-management intervention in the future. Beaver presented the results of a qualitative study, exploring patient experiences of neo-adjuvant chemotherapy for breast cancer. The sample included a relatively young group of women (mean age 49 years), many of whom had young children and/ or were caring for elderly parents. The main themes that emerged from the data included coping with the rapid transition from ‘well’ to ‘ill’, the challenges of processing complex information, perceived lack of emotional support, needing empathy, impact on family, regaining control, and creating a new ‘normal’. The women in this study were able to identify key timepoints when information and support would have been beneficial. This information is vital in developing services and interventions that will meet the complex needs of these patients and potentially prevent hospital admissions and late referral to psychological services.

## Management of toxicities related to chemotherapy and targeted therapy

Pharmacology, the science of drug actions, studies the pharmacokinetics and pharmacodynamics of therapeutic agents. Pharmacokinetic parameters are the absorbance, metabolism, distribution and excretion of drugs, while pharmacodynamic studies concentrate on the interaction between the drug and its target cells and tissues and the body’s response to that interaction. Cardiotoxicity is one of the most significant adverse effects of cancer treatment and is responsible for considerable morbidity and mortality. The most frequent clinical manifestation of cardiotoxicity is asymptomatic or symptomatic left ventricular dysfunction. It may be induced not only by conventional cancer therapies, like anthracyclines, but also by new antitumoural targeted therapies such as trastuzumab. Atay reported that cardioprotective strategy protocols should be developed for patients undergoing treatment. Although cardiotoxic effects of cancer treatment occur infrequently, early detection and toxicity require cardiac monitoring. Unfortunately, no proven strategies are available. The American Heart Association recommended close monitoring of cardiac function during anthracycline treatment but does not specify how often or by which means. Nurses can also minimise the risk of cardiac toxicity by understanding the types and doses of chemotherapy or targeted therapy that patients have received previously, and whether patients have received radiation therapy to the chest. Knowledge about patients’ previous exposure to chemotherapy could help nurses alleviate potential risks associated with treatment administered in the metastatic setting. No specific evidence-based guidelines exist for the management of chemotherapy-induced cardiac dysfunction. The Heart Failure Society of America’s practice guidelines state that before treatment, interventions should be employed to control the following cardiovascular risk factors: body weight, hypertension, hyperglycaemia, smoking, and alcohol consumption. Clinical studies have demonstrated the benefit of angiotensin-converting-enzyme (ACE) inhibitors and beta-blockers in patients with chemotherapy-induced cardiac dysfunction. Dielenseger presented the interesting issue of caring for patients with skin toxicity. Skin toxicity is a common issue with wide consequences, including the management of the symptoms themselves, but also encompassing issues of body image and social interactions. Health professionals are not focused on prevention for the moment, but this should be an aim in the near future.

## Hope across cancer care

Hope is closely linked to hopelessness, and cancer diagnoses can cause emotional chaos and a feeling that one has lost control over life. Hopelessness is a common reaction to a diagnosis of cancer. However, precisely because hope is a part of life, hope is present in cancer patients. Hammer from Denmark presented a study carried out at a surgical unit at a Danish university hospital with the aim of quantifying and understanding hope. The data collection was based both on interviews and drawings. The findings demonstrated the need for nurses and other healthcare professionals to support patients finding hope, starting from the time of diagnosis. Nurses who meet cancer patients in palliative care face a great challenge when supporting hope in patients and their families. In that context, Benzein reported her presentation about nurses’ perspective of hope in cancer care. The results from the previous study showed that nurses view hope as an inner strength and energy in their patients. The nurses argued one of the most important sources of hope that cancer patients rely on are their friends and relatives. Nurses can also be important sources of support and hope provision for patients with cancer and their families. By being aware of their own perceptions of hope, nurses can open up space to invite patients and families to narrate their experiences and needs in order to experience hope. The nurse–patient/family relationship should be characterised by a partnership in which hope can be created in a collaborative way. Hope can be a valuable asset in coping with terminal disease, and it has been defined as ‘a confident yet uncertain expectation of achieving a future good that, to the hoping person, is realistically possible and personally significant’. In this landscape, Van Laarhoven from the Netherlands involved the audience, speaking about hope and end-of-life care for cancer patients.

## Survivorship and follow-up

Evidence for the health benefits of physical activity and exercise for cancer survivors is accumulating. However, the meaningfulness of physical activity from the perspective of the patient has not yet been established. Midtgaard from Denmark presented the results of a meta-synthesis of qualitative research about the meaningfulness of physical activity and exercise in cancer rehabilitation, where 61 papers were critically appraised and 17 papers were identified for inclusion. The findings of this meta-synthesis suggested that cancer survivors experience physical activity and exercise as a means to fulfil their mental, social, and physical well-being independent of disease status. In addition to the current evidence on the efficacy of exercise training in cancer survivorship, it is incumbent upon clinicians and policy-makers to acknowledge and promote the meaningfulness of physical activity and exercise and to use this knowledge to provide new solutions to current problems related to recruitment, adherence, and implementation. Kaasa returned to the theme of technology aids in supportive cancer care, identifying a need for using tools that combine symptom assessments and classification, evidence-based guidelines, and decision-making support. These tools should be computerised since this will allow individualised and dynamic symptom assessment, thus improving the efficiency of consultations. The overall aim by applying the software to clinical practice is to enhance the patient-centred communication and treatment by facilitating an optimal exchange of information between the patients and the healthcare providers. Pravettoni of Italy pointed to the need to find efficient strategies to foster health promotion and a sensitisation campaign for job reintegration among both cancer survivors, organisational management and society, in order to facilitate and improve the empowerment and well-being of cancer survivors.

## Cancer surgery and nursing

Current issues related to surgery and nursing were discussed during this joint session between EONS and ESO. Cardoso, surgeon from Portugal, outlined the main critical points of oncoplastic surgery, especially relating to breast cancer surgery. Aesthetic results from breast-conserving surgery are in 10%–30% of cases considered fair or even poor. Increasing sophistication, the need for improved global outcomes, and patients’ demand of a better aesthetic outcome have led to the development of oncoplastic surgery. This involves simultaneous excision of the tumour and reconstruction of the defect, applying techniques used frequently in cosmetic breast surgery. Oncoplastic surgery is becoming progressively available in centres dedicated to breast cancer treatment all over the world. However, the monitoring of oncological and aesthetic results has not come hand in hand with the dissemination of these new techniques. A better outcome for patients with oncoplastic surgery is expected, but we must support our findings and further progression with the publication of results – ideally, from clinical trials. Another topic of great current interest was presented by Lichosik, nurse coordinator of the Robotic Surgery School in Milan. She explained the importance of the nurses’ role in robotic surgery. The professional nursing staff is responsible for following best practice rules and to periodically analyse roles and habits. Robotic surgery could be an effective instrument, improving everyday practice. The role of robotics nurse specialist is both challenging and exciting because the technology is so new, and the role is open to interpretation and definition. The experience of the European Institute of Oncology in Milan also showed the needs of continuous education, especially regarding nursing skills and the creation and revision of guidelines and specific protocols. An amazing presentation of surgical oncology in the elderly was performed by the surgeon Audisio from the United Kingdom. He emphasised that it is very important to accept the diversity of the older individual. As geriatric individuals may suffer from issues such as cognitive impairment or delirium, it is crucially important that medical professionals understand these differences and their impact, especially when obtaining informed consent. Social and familiar support needs to be taken into account when offering active treatment to the elderly or simply arranging for imaging or follow-up visits.

## Conclusion

Remaining closely within the nursing field, the clinical mindset was identified as an important factor in nursing practice, which must be supported by a policy of multidisciplinary oncology nursing research. This ECCO Congress plays a crucial role in raising the awareness of the oncology community in relation to new progresses in diagnosis and treatment. We must consider that the degree to which this awareness can be put into daily practice across Europe is dependent on different factors: politics, funding, commercial partnership, and culture. This meeting represents a unique occasion for cancer nurses to share experiences, to be inspired and to inspire, as well as creating and strengthening the global nursing network. The multidisciplinary approach is the way of the future for healthcare professionals to better respond to the growing need to have personalised and targeted care. The European societies are starting to place the individual human being with specific treatment needs at the centre of cancer research, instead of centring the treatment itself. Hence, the role of nursing is increasingly central, and collaboration with other healthcare professionals is vital to achieve the goal of better responding to the needs of society.
